# Two-Birds-with-One-Stone Synthesis of Hydrophilic and Hydrophobic Fluorescent Carbon Nanodots from *Dunaliella salina* Biomass as 4-Nitrophenol Nanoprobes Based on Inner Filter Effect and First Derivative Redshift of Emission Band

**DOI:** 10.3390/nano13101689

**Published:** 2023-05-21

**Authors:** Thomais A. Skolariki, Theodoros G. Chatzimitakos, Lamprini Sygellou, Constantine D. Stalikas

**Affiliations:** 1 Laboratory of Analytical Chemistry, Department of Chemistry, University of Ioannina, 45110 Ioannina, Greece; thomiskolariki@windowslive.com (T.A.S.); chatzimitakos@outlook.com (T.G.C.); 2 Foundation for Research and Technology Hellas/Institute of Chemical Engineering Sciences (FORTH/ICE-HT), Stadiou Str., P.O. Box 1414, 26504 Rio-Patras, Greece; sygellou@iceht.forth.gr

**Keywords:** hydrophilic/hydrophobic carbon nanodots, 4-nitrophenol fluorescent sensing, *Dunaliella salina*, quenching mechanism, fluorescence band redshift

## Abstract

4-Nitrophenol (4-NP) has been listed as a priority pollutant and has also been reported as a human urinary metabolite used as a marker to evaluate exposure to certain pesticides. In the work herein, a solvothermal approach is applied to the one-pot synthesis of both hydrophilic and hydrophobic fluorescent carbon nanodots (CNDs), utilizing the halophilic microalgae *Dunaliella salina* as a biomass precursor. Both kinds of the produced CNDs showed appreciable optical properties and quantum yields, good photostability and they were capable of probing 4-NP by quenching their fluorescence through the inner filter effect. Interestingly, a prominent 4-NP concentration-dependent redshift of the corresponding emission band of the hydrophilic CNDs was noticed, which was further exploited, for the first time, as an analytical platform. Capitalizing on these properties, analytical methods were developed and applied to a variety of matrixes, such as tap water, treated municipal wastewater and human urine. The method based on the hydrophilic CNDs (λ_ex_/λ_em_: 330/420 nm) was linear in the range of 0.80–45.0 μM and showed acceptable recoveries (from 102.2 to 113.7%) with relative standard deviations of 2.1% (intra-day) and 2.8% (inter-day) for the quenching-based detection mode and 2.9% (intra-day) and 3.5% (inter-day) for the redshift one. The method based on the hydrophobic CNDs (λ_ex_/λ_em_: 380/465 nm) was linear in the range of 1.4–23.0 μM, with recoveries laying within the range of 98.2–104.5% and relative standard deviations of 3.3% and 4.0% for intra-day and inter-day assays, respectively.

## 1. Introduction

Nitrophenols are well-known hazardous substances with high solubility in water and low degradability in soil and groundwater [1]. The 4-nitrophenol (4-NP) is widely used in the production of pharmaceuticals and pesticides, such as methyl and ethyl parathion. It is also used as a leather fungicide and acid-base indicator [2,3]. Toxicity studies have shown that 4-NP can cause respiratory depression with cyanosis, headaches, drowsiness and nausea in humans, and dermal irritation or lethality in other organisms, such as rabbits and mice [4]. For these reasons, 4-NP has been listed as a potential carcinogen, teratogen and mutagen [2,5]. In addition, 4-NP is a major urinary metabolite, which can be used as a biological marker to indicate exposure to various pesticides, or is produced by particular enzymes, if the corresponding substrates are present [6,7,8]. To date, a variety of analytical methods have been reported, such as chemiluminescence [9], spectrophotometry [10], spectrofluorometry [11,12], capillary electrophoresis [13], gas chromatography–mass spectrometry [14,15] and liquid chromatography [16,17]. Some of these methods are tedious and time-consuming; as a result, monitoring 4-NP with fast, easy and sensitive methods is favorable.

Lately, fluorescent carbon nanodots (CNDs) have attracted increasing interest due to many advantages, such as small size, high photostability, cheap and easily available precursors, good biocompatibility and low cytotoxicity [18,19]. CNDs are quasi-spherical nanoparticles with sizes below 10 nm, containing several functional groups on their surface, as well as doping elements [20,21]. To date, CNDs have been used as analytical probes for the detection of environmental pollutants, food safety, etc. [22,23].

Synthetic methods, in conjunction with pure chemicals or natural precursors, improve the chemical diversity and the properties of the CNDs. Many natural precursors have been used, such as orange and lemon peels, apple seeds, human fingernails, etc. [24,25,26,27]. Natural resources and raw materials provide a viable option of renewable materials with low cost and good biocompatibility [28]. The solvothermal approaches that use cheap and “green” precursors have garnered more attention because of the low energy needs and the easy control of the reaction [26,29,30].

*Dunaliella salina* is a halotolerant green alga known for producing carotenoids and glycerin under certain conditions. Due to its halophilic nature, *D. salina* can survive environmental changes, such as high salinity, low temperature, high irradiance and nutrient limitations. It can be used for carotenoid extraction, as a food coloring factor and lately, as a natural precursor for nanoparticle synthesis [31,32,33]. To date, the use of *D. salina* has been reported for the synthesis of hydrophilic CNDs, which were applied as a selective fluorescence nanoprobe for the detection of Fe(III) in water samples, as radical scavengers [34] and as sun protection filters with an enhanced sun protection factor [35]. Therefore, it holds promise as a natural source to produce CNDs.

In this study, a facile solvothermal approach is employed for the synthesis of two kinds of CNDs, using the algal biomass of *D. salina* as a natural precursor. Lipids, proteins, carbohydrates, glycerol and carotene are some of the main components or classes of components of this kind of biomass. Based on this composition, hydrophilic and hydrophobic CNDs were able to be produced by applying a one-pot procedure, in addition to an already reported method of synthesizing CNDs from *D. salina* [34]. Both of CNDs were utilized as 4-NP probes for wastewater, tap water and human urine analysis. The fluorescence of the CNDs was effectively quenched by 4-NP, exhibiting good sensitivity. What is more, a prominent concentration-dependent redshift of the corresponding emission band was noticeable as the concentration of 4-NP increased. This spectroscopic feature was harnessed for the first time in this study, and its potential for being used as an alternative quantification platform is further evaluated.

## 2. Materials and Methods

### 2.1. Chemicals

All the chemicals used were at least of analytical grade. Toluene was purchased from Honeywell (Honeywell, Charlotte, NC, USA). 4-nitrophenol, 2-nitrophenol, 3-nitrophenol, 2-methyl-4,6-dinitrophenol, 2,4-dichlorophenol, 2,3,5-trichrolophenol, humic acids, ammonia solution, ammonium chloride, sodium chloride and sodium sulfate were purchased from Sigma-Aldrich (Sigma-Aldrich, Steinheim, Germany). Quinine hemisulfate monohydrate (98%, suitable for fluorescence) was purchased from Alfa Aesar (Alfa Aesar, Karlsruhe, Germany). Stock metal solutions (Mn^2+^, Fe^3+^, Ag^+^, As^5+^, Se^4+^, Cr^3+^, Cu^2+^, Co^2+^, Ni^2+^, Li^+^, Cd^2+^, K^+^, Na^+^, Hg^2+^ and Ca^2+^) were obtained from Merck (Merck, Darmstadt, Germany). Amino acids (lysine, methionine, phenylalanine, leucine, histidine, arginine, tyrosine, threonine, proline, serine, glycine, tryptophan, asparagine, glutamine, cysteine, alanine and valine) were acquired from Fluka (Fluka, Seelze, Germany). Stock solutions of 4-NP were prepared in amber glass vials and stored at room temperature. All the experiments were conducted using double distilled water (DDW).

### 2.2. Culture Conditions

The strain of *D. salina* used as a precursor was the Duna 30, secluded from the salt works of Megalo Emvolo (North Greece, Aegean Sea). The cultivation required quadruplicate batches (5 L volume) of mildly aerated (0.5 vvm) synthetic seawater (Tropical Marine™ salts dissolved in deionized water, (Wartenberg, Germany)) of 120 ppt salinity at a temperature of 26 ± 1 °C, a photoperiod of 12:12 h L: D, 100 µmol photons m^−2^·s^−1^. The seawater was enriched with 1 mL of N-free Conway medium and 0.1 mL of Conway medium vitamin stock. The strain was cultivated until senescence (about 7 days, on average).

### 2.3. Synthesis of CNDs from Dunaliella salina

The homogenous green dry powder of *D. salina* (80 mg) was dispersed in toluene (40 mL) through ultrasonication for 15 min. The mixture was transferred to a Teflon-lined autoclave and heated at 200 °C for 5 h, and then it was left to cool to room temperature. The obtained mixture was centrifuged at 8000 rpm for 5 min to separate the hydrophobic CND (HB-CNDs)-containing organic phase from the precipitate, where the hydrophilic CNDs (HL-CNDs) laid. Then, the toluene phase was evaporated to dryness to obtain the HB-CNDs, as a residue. The dark brown precipitate obtained after centrifugation was washed three times with toluene and with ethanol until the after-washing solvents emitted no fluorescence. The ethanol was removed from the residue via evaporation and the dried precipitate was dissolved in DDW and dialyzed against water for 24 h using a dialysis tubing membrane (MWCO: 1000 Da). After freeze drying, a yellow powder of HL-CNDs was collected, which was stored in the dark until use.

### 2.4. Instrumentation and Characterization

Details are given in the Supplementary Materials.

### 2.5. Quantum Yield (QY)

Details are given in the Supplementary Materials.

### 2.6. Probing 4-NP with Hydrophilic and Hydrophobic CNDs

For the determination of 4-NP using the HL-CNDs, 2.5 mL of a sample were transferred to a glass vial followed by the addition of 0.25 mL of Britton–Robinson buffer (0.1 M, pH = 9.5) and 0.25 mL of an aqueous CND solution (1 mg/mL). After vortexing, the photoluminescence (PL) intensity of the CNDs was measured at 330/420 nm (λ_ex_/λ_em_). Moreover, the red shift of the emission peak wavelength was recorded after excitation at 330 nm and first derivative transformation was carried out.

For the determination of 4-NP using the HB-CNDs, 5 mL of a sample containing 0.25 mL of Britton–Robinson buffer (0.1 M, pH = 5.5) was extracted with 3 × 1.0 mL of ethyl acetate (vortexing for 30 sec and letting it stand for 1 min, for layer separation). The upper phases were collected and transferred to another vial, where 20 μL of an HB-CND solution (5 mg dissolved in 1 mL of ethyl acetate) was added. After vortexing, the PL intensity of the HB-CNDs solution was measured at 380/465 nm (λ_ex_/λ_em_). Plots were built using the ratio *F*_0_/*F* of the CNDs, where *F*_0_ is the fluorescence of the sample in the absence of 4-NP and *F* is the fluorescence of the sample in the presence of 4-NP.

### 2.7. Sample Preparation

Tap water was obtained from the urban area of Ioannina, Greece and was analyzed directly without any pretreatment. Municipal wastewater was sampled from the wastewater treatment plant of Ioannina, Greece. The sample was filtered via a Whatman paper filter to remove particulate matter from the sample, and then it was further analyzed. Human urine samples were collected from a healthy volunteer aged 25 years. The samples were centrifuged with a 2 mL VIVASPIN concentrator (Membrane: 300.000 MWCO, VIVASCIENCE, Hannover, Germany) at 8000 rpm, for 10 min and were diluted 10 times, before analysis with HL-CNDs. For the same matrix, only dilution was needed when HB-CNDs were used, obviating the ultrafiltration step.

## 3. Results and Discussion

### 3.1. Characterization of CNDs

**Figure 1:**
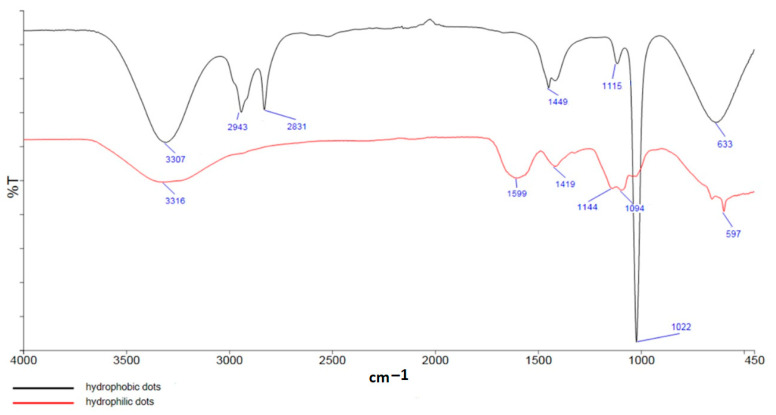
FTIR spectra of HB-CNDs (black line) and HL-CNDS (red line).

The FTIR spectra of the synthesized CNDs (Figure 1) show a broad band around 3310 cm^−1^ attributed to the stretching vibrations of the O–H and N–H groups, while the broad peaks in the FTIR spectrum of HB-CNDs around 2943 cm^−1^ and 2832 cm^−1^ account for the stretching vibrations of the CH and CH_2_ aliphatic stretching groups. The peak at 1026 cm^−1^ is attributed to the stretching vibrations of the C–O–C and C–O groups, while those at 1144 cm^−1^ and at 1419 cm^−1^ can be ascribed to the C–O and C–N bonds. Finally, the peaks at ~1600 cm^−1^ confirm the existence of the –COO^−^ asymmetric and symmetric stretching vibrations [20,22,23,25].

**Figure 2:**
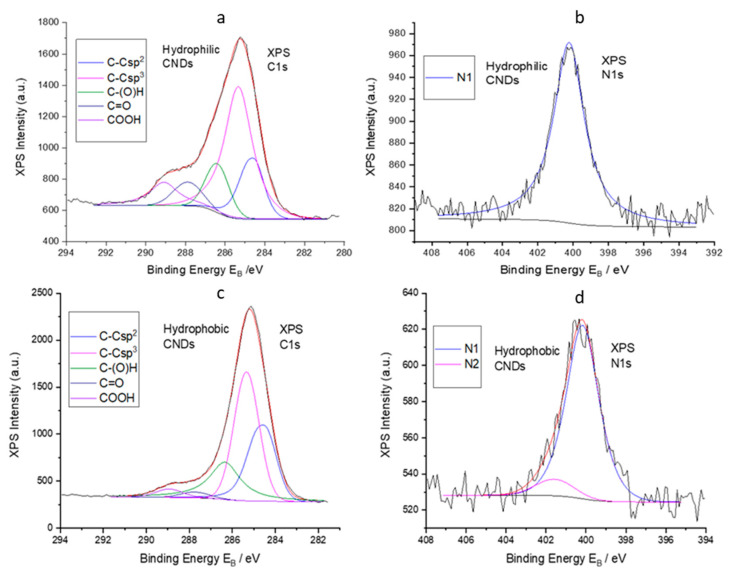
Deconvoluted XPS peaks of C1s (**a**) and N1s (**b**) of HL-CNDs and deconvoluted XPS peaks of C1s (**c**) and N1s (**d**) of HB-CNDs.

The XPS spectra were acquired in order to identify the chemical state of the elements on the surface of the CNDs and the elemental composition. Figure 2 shows the deconvoluted XPS C1s and N1s peaks for the HL-CNDs and HB-CNDs. The first peak, C1s, consists of the following components (Figure 2a,c): C–C with sp^2^ (C=C) and sp^3^ (C–C) configuration; hydroxyls or epoxides (C–O(H)); carbonyls (C=O) and carboxyls (O=C–OH) [36]. The second peak, N1s (Figure 2b,d), consists of one or two components at binding energies of 400 ± 0.1 eV (N1) and 401.6 ± 0.1 eV (N2) associated with C–NH–C=O and positively charged nitrogen atoms (–NH_3_^+^), confirming the existence of nitrogen doping in various configurations. The % of the carbon and nitrogen components and the % of the atomic concentration of C, O and N of the CNDs are given in Table S1.

The zeta potentials (*ζ*) of HL-CNDs and HB-CNDs in DDW and ethyl acetate, respectively, were determined to be −17.0 mV and −0.54 mV, respectively. The negative value of the HL-CNDs implies that the hydroxyl and carboxyl groups dominate in the charged CNDs. The particle size distribution of both materials down to the nano range is portrayed in Figures S1 and S2. The mean hydrodynamic diameters of the hydrophilic and hydrophobic nanoparticles are 20 nm and 28 nm, respectively, while the TEM images showed sizes of around 4 nm.

### 3.2. Optical Properties of CNDs

The UV-Vis spectra of the synthesized HL-CNDs and HB-CNDs, given in Figure S3, exhibit broad absorption bands at around 270 nm and 280 nm, due to the n-π* and π-π* transition of the –C=O bonds of the carboxyl groups and aromatic sp^2^ (–C=C–) domains, respectively. The absorption shoulder in the spectrum of the HB-CNDs extending from 390 to 430 nm is attributed to the n-π* transitions of the aromatic C=C bonds and non-bonding electrons of the oxygen atoms in the C=O or C-O bonds [37]. The dispersions of the CNDs in water or organic solvent are yellow-colored under daylight and blue-colored under UV light irradiation.

**Figure 3:**
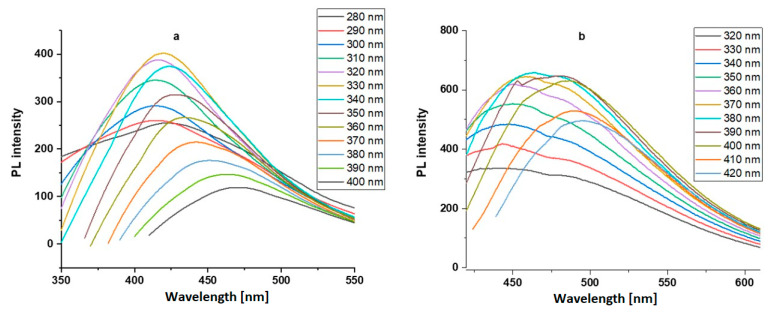
Emission spectra of (**a**) HL-CNDs in DDW and (**b**) HB-CNDs in ethyl acetate.

The PL properties of the CNDs were examined, and the emission spectra were recorded at different excitation wavelengths (Figure 3). Both kinds of CNDs exhibited excitation-dependent fluorescence emission with a red shift of peak wavelength recorded as the excitation wavelength increased. The HL-CNDs showed a maximum emission at 420 nm when excited at 330 nm. The QY of the HL-CNDs was found to be 9%. Similarly, the HB-CNDs showed a maximum emission at 465 nm when excited at 380 nm, and the QY in ethyl acetate was found to be 13%.

### 3.3. CND Fluorescence Stability

**Figure 4:**
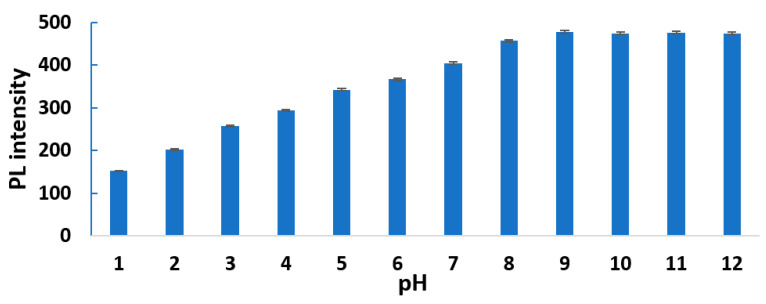
PL intensity of HL-CND solution at various pH values as a function of pH, at λ_ex_ = 330 nm and λ_em_ = 420 nm. Error bars represent the standard deviation of three replicates.

First, the PL intensity of the HL-CNDs was examined in aqueous solutions with the pH varying up to 12.0. As can be seen in Figure 4, there is an increase in the PL intensity as the pH of the solution increases from 1.0 to 9.0, acquiring a constant value at pH = 12.0. This variation of the PL intensity can be attributed to the protonation/deprotonation of the functional groups on the surface of the HL-CNDs, such as the carboxyl and amino groups [37,38]. Next, the effect of ionic strength on the PL intensity was investigated. No change in the PL intensity were recorded in the presence of sodium chloride and sodium sulfate, both being at concentrations up to 1.0 M.

The stability of the PL intensity under constant irradiation was also examined, using a 150 W Xe lamp. The PL intensity was found to be stable for both kinds of CNDs after 1 h of irradiation. Finally, after 1 month of storage at 4 °C in the respective solvents, no decrease in the PL intensity was recorded for the CND solutions (intensity decrease: <0.1%).

### 3.4. Optimization of 4-NP Probing-Selectivity Study

The first step in the optimization part was to examine the pH effect on the quenching of the HL-CNDs by the ionizable 4-NP. As can be seen in Figure S4, the quenching of the HL-CNDs fluorescence increases as the pH of the solution increases in the pH range of 2.0–9.0, and then decreases. Although the PL change is not significant, all subsequent experiments were carried out at pH 9.0 in order to achieve the maximum response. Additionally, the effect of ionic strength and the presence of humic acids on the PL quenching caused by 4-NP were investigated. Neither ionic strength up to 1.0 M nor humic acids up to 10 mg/L had any effect on the PL quenching.

The detection of 4-NP with the HB-CNDs was carried out in an organic solvent due to the poor dispersibility in water. Because of this limitation, 4-NP was extracted from the sample matrix into an organic solvent. Toluene, chloroform, hexane and ethyl acetate were examined for their potential to extract 4-NP without affecting the fluorescence properties of CNDs. The maximum extraction efficiency was achieved by applying a simple liquid–liquid extraction with a low volume of ethyl acetate (Figure S5) when the pH was adjusted to ~5.5 (Figure S6).

**Figure 5:**
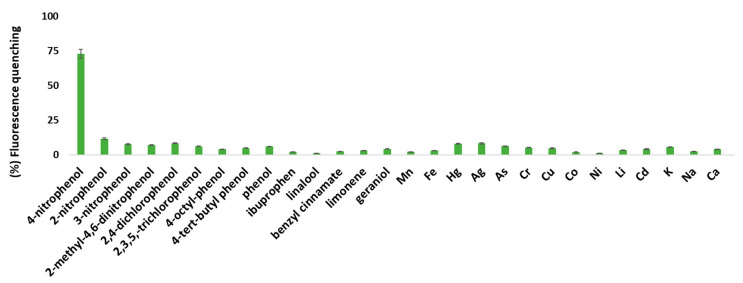
PL quenching of HL-CNDs caused by phenols, allergens, pharmaceutical compounds and metal ions, at concentrations of 50 μΜ at λ_ex_ = 330 nm and λ_em_ = 420 nm. Error bars represent the standard deviation of three replicates.

With the aim to explore the selectivity of the two PL probes, potential interferences were tested, such as phenols, metal ions, pharmaceutical compounds and allergens (50 μΜ), as potential quenchers of the CNDs. In Figure 5, it can be seen that 4-NP causes an appreciable quenching on the PL, whereas other compounds have little effect at the concentrations studied, except 2-nitrophenol, Hg and Ag, which cause quenching of 11.8, 8.1 and 8.3%, respectively. This bespeaks a response of the synthesized CNDs to 4-NP under certain conditions, leading to the development of a fairly selective PL nanoprobe. On the other hand, the quenching on the fluorescence of the HB-CNDs in ethyl acetate by the different ions and molecules tested was even lower than that recorded in the HL-CNDs (data not shown).

### 3.5. Probing 4-NP Using HL-CNDs

**Figure 6:**
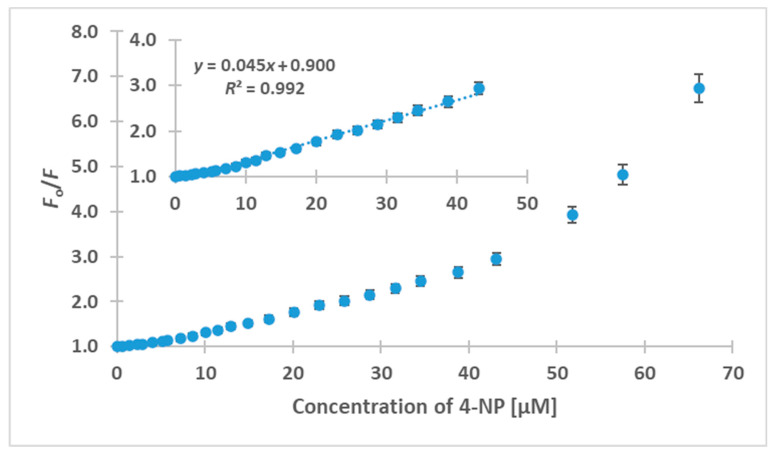
Plot of fluorescence quenching of the HL-CNDs versus 4-NP concentration at λ_ex_ = 330 nm and λ_em_ = 420 nm. Inset: linear calibration curve in the concentration range of 0.8 to 45.0 μM. Error bars represent the standard deviation of three replicates.

The fluorescence emission spectra of the HL-CNDs at various 4-NP concentrations can be seen in Figure S7. It is obvious that a decrease in the emission maximum is accompanied by an unexpected red shift of the emission peak wavelength. Figure 6 shows the ratio of *F*_0_/*F* at a fixed λ_ex_/λ_em_ of 330/420 nm for concentrations of 4-NP up to 66.0 μM. A linear response was recorded under optimum conditions for 4-NP concentrations between 0.80 and 45.0 μΜ, with *R*^2^ = 0.992. The limit of detection (LOD, calculated as the 3 × signal-to-noise ratio) was found to be 0.30 μM, and the relative standard deviation (% RSD) for three measurements of 4-NP at 20 μM was 2.1% for intra-day and 2.9% for inter-day assays. The recovery experiments were carried out using tap water, effluent and human urine after spiking the relevant matrixes with 5.1, 15.3 and 40.2 μΜ. As can be seen in Table 1, the recoveries ranged between 102.2% and 103.9% for tap water and between 103.0% and 107.8% for effluent, while for human urine, they ranged between 102.2 and 113.7%. All the above corroborate acceptable analytical figures of merit for the analytical method.

### 3.6. Quenching Mechanism of the HL-CNDs

To gain further information on the mechanism that governs the quenching of the HL-CNDs from 4-NP, relevant experiments were carried out. First, the quenching caused by various concentrations of 4-NP was examined at different temperatures (i.e., 20, 30 and 40 °C). A minor decrease in the quenching was observed as the temperature increased (data not shown), hinting at a static mechanism owing to a weak interaction of the electron-rich amino group of N-CDs with the electron-deficient nitroaromatic 4-NP. However, since the observed differences were minor, static is not the principal quenching mechanism. To further verify the occurrence of static quenching, the absorbance spectra of a HL-CNDs solution, a 4-NP solution and their mixture were recorded (Figure S8). This spectrum was compared with the theoretical spectra, which are the algebraic summation of the individual spectra. A decrease in the absorption spectrum of the mixture solution was recorded, bespeaking the formation of a complex [25].

Next, the absorption spectrum of 4-NP, along with the excitation and emission spectra of the HL-CNDs, were depicted. As can be seen in Figure S9, an overlap between the absorption spectrum of 4-NP and the excitation spectrum of the HL-CNDs is observed, suggesting an inner filter effect (IFE) mechanism [39].

### 3.7. Harnessing the Shift of Emission Band of HL-CNDs in the Presence of 4-NP

**Figure 7:**
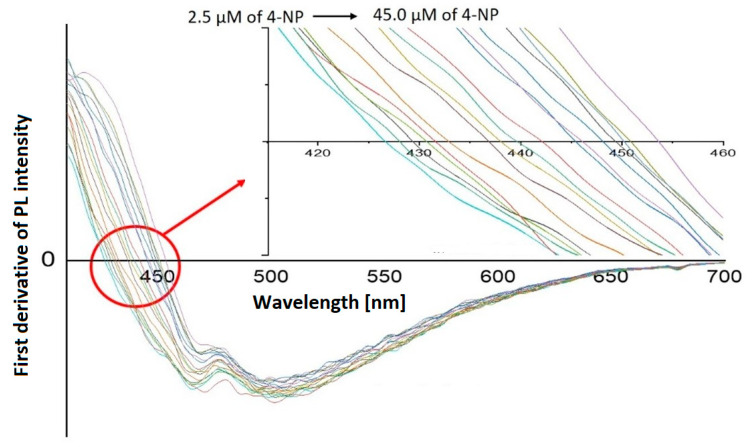
First derivatives of PL spectra at concentrations of 4-NP from 2.5 μM to 45.0 μM, under optimum conditions at λ_ex_ = 330 nm. Inset: close-up of the *x*-axis (wavelength) where first derivatives are zeroed.

**Figure 8:**
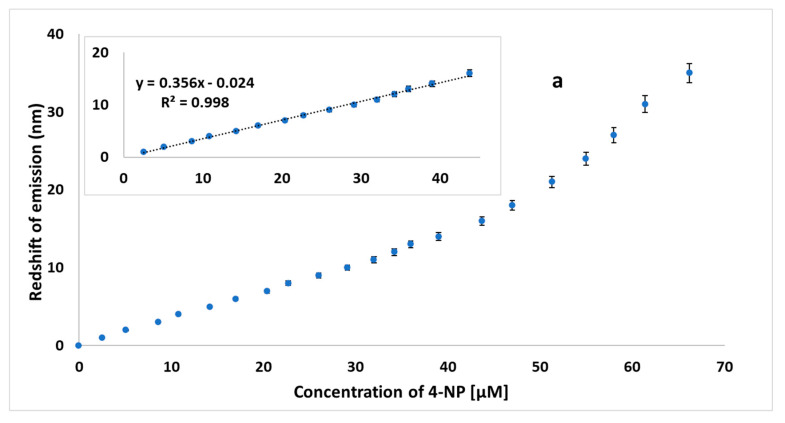

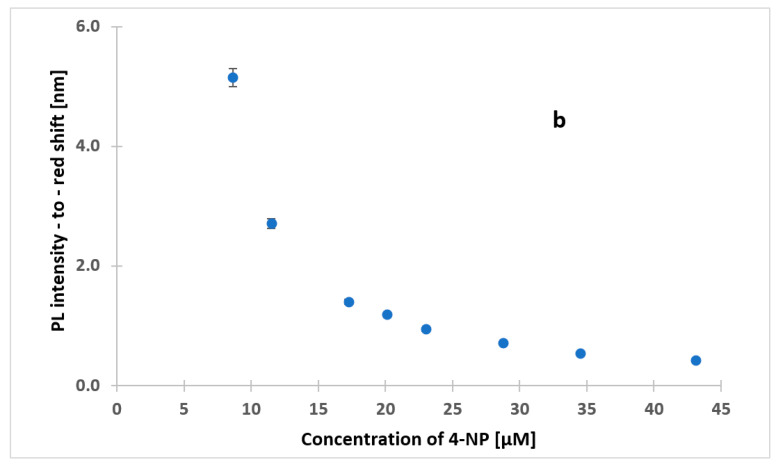
(**a**) Zero values of the first derivatives of emission spectra as a function of concentration of 4-NP (μΜ). Inset: linear range at concentrations between 2.5 and 45 μM and (**b**) ratio of PL intensity-to-emission red shift (nm) at different concentrations of 4-NP, using HL-CNDs (λ_ex_ = 330 nm). Error bars represent the standard deviation of three replicates.

The addition of 4-NP to HL-CNDs caused not only a quenching to the fluorescence intensity, but also a prominent redshift in the emission spectrum, as mentioned above. As the concentration increased to 55.0 μΜ, this shift added up to a total of 35 nm. The gradual red shift of the fluorescence maximum emission versus the quencher concentration has rarely been reported for other fluorescence systems [40]. In our case, it is noteworthy that only 4-NP causes this unpredicted redshift, thus enabling the quantification of 4-NP in the presence of other potential quenchers, such as 2-NP, Hg and Ag. Motivated by this behavior, we examined whether the observed redshift could be used for 4-NP detection. To this end, the emission spectra of the HL-CNDs in the presence of 4-NP at various concentrations were recorded, and a first derivative transformation was carried out. As shown in Figure 7, the redshift, i.e., the wavelength where the first derivative is zeroed, is closely related to the concentration of 4-NP. A linear response was observed in the range of 2.5–45.0 μM for 4-NP, as portrayed in Figure 8a, with *R*^2^ = 0.998, suggesting a high degree of linearity. The % RSD for three measurements of 4-NP at 20.0 μM was 2.8% for intra-day and 3.5% for inter-day assays.

Alternatively, a plot of the ratio of PL intensity-to-red shift (nm) versus the concentration of 4-NP was drawn (Figure 8b). From its pattern and the gradient of the slope along this curve, it was evident that at low concentrations of 4-NP, the rate of variation of this ratio was markedly higher than that in the rest of the curve. Practically, this signifies that the fluorescence quenching at low concentrations is higher than the redshift itself, while this behavior is reversed as the concentration increases (>16 μΜ).

Mixtures containing 4-NP and 2-NP at molar ratios of up to 1:20 (4-NP: 2-NP) showed no interference at all with the assay of 4-NP. Finally, recovery experiments from spiked wastewater at 4-NP concentrations of 7, 15 and 25 μM were also carried out, and the recorded recoveries were found to be in the range of 104.6–111.6%, suggesting satisfactory accuracy. The proposed redshift method can be applied either as a standalone one or in connection with one of the other two already described, for the purpose of unequivocal confirmation of the presence of 4-NP.

### 3.8. Probing 4-NP Using HB-CNDs

**Figure 9:**
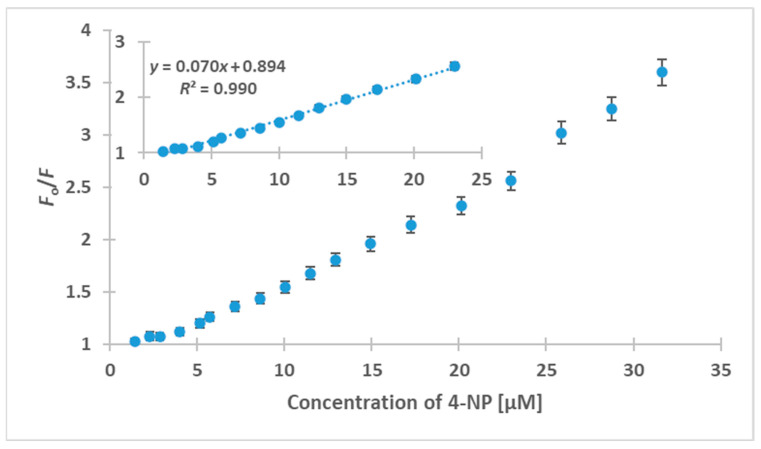
Plot of fluorescence quenching of the HB-CNDs versus 4-NP concentration at λ_ex_ = 380 nm and λ_em_ = 465 nm. Inset: linear calibration curve in the range of 1.4 to 23.0 μM. Error bars represent the standard deviation of three replicates.

An analytical probe was developed, taking into account that considerable fluorescence quenching is caused on HB-CNDs by 4-NP. Figure 9 shows the ratio *F*_0_/*F* of the CND fluorescence in the absence, *F*_0_, and the presence of 4-NP, *F*, at concentrations up to 32.0 μM. A linear response was recorded for 4-NP concentrations in the range of 1.4–23.0 μM, with a coefficient of determination, *R*^2^ = 0.990. The LOD was found to be 0.5 μΜ, and the relative standard deviation (% RSD) for three measurements of a 4-NP solution of 10 μM was 3.3% for intra-day and 4.0% for inter-day assays. Next, recovery experiments were carried out on testing samples from a wastewater treatment plant and a human urine sample, spiked with 4-NP at concentrations of 7.5, 15.5 and 22.5 μΜ. The results of three replicate analyses are shown in Table 2. Acceptable analytical figures were recorded in all cases, with the recoveries for the effluent ranging between 98.2% and 104.5%, while for human urine, they were between 99.3% and 104.0%. It is noteworthy that the urine sample was analyzed after extraction without any step to precede for the removal of high-molecular weight compounds present in the matrix.

### 3.9. Comparison with Other CND-Based Methods

A comparison was made in terms of the materials used and the major analytical characteristics, such as the linear range and the recoveries. As can be seen from Table S2, the precursor material used herein for the synthesis of the CNDs is cheap and derived from natural resources. The developed methods provide broad applicability by determining 4-NP not only in complex aqueous matrixes, but also in biological matrixes, such as human urine. The analytical figures of merit of the methods are comparable with the previously reported methods which use different precursors and matrixes, although the analysis time and RSD are fairly low in our case. Moreover, the redshift fluorescence method based on HL-CNDs exhibit high recoveries, good linearity and satisfactory RSD values. Finally, it provides a novel analytical platform, which can be applied more effectively when 2-NP or other potential interferences are present at high concentrations in comparison to 4-NP.

## 4. Conclusions

Two kinds of CNDs were successfully synthesized by a solvothermal approach, using *Dunaliella salina* biomass as a green precursor. Both HL-CNDs and HB-CNDs can be used as nanoprobes for the detection of 4-NP. The developed CND probes for the detection of 4-NP in the water samples and human urine were based on the IFE quenching mechanism. Moreover, a prominent concentration-dependent emission redshift of the HL-CNDs was harnessed, for the first time, as an additional fluorescent platform for the determination of 4-NP. Although figures of merit, such as the LOD and the linear range of the latter are inferior to the other two studied approaches, it can provide a new analytical prospect of fluorescence in chemical analysis under certain conditions. It is interesting that even the naturally occurring *Duna 30* produced CNDs with the same analytical characteristics (data not reported). However, *D. Salina* from natural habitats has some restrictions, such as limited availability considering the short period of harvesting over the year and scarcity of sites where it occurs.

## Figures and Tables

**Table 1:** Analysis of real samples and recoveries of 4-NP from different matrixes using the fluorescent HL-CNDs.

Sample	4-NP Concentration (μΜ)		Recovery (%)
	Added	Determined	
Tap water	0	non-detected	-
	5.1	5.3	103.9
	15.3	15.8	103.3
	40.2	41.1	102.2
Effluent from a wastewater treatment plant	0	non-detected	-
	5.1	5.5	107.8
	15.3	16	104.6
	40.2	41.4	103.0
	0	non-detected	-
	5.1	5.8	113.7
Human urine	15.3	16.1	105.2
	40.2	41.1	102.2

**Table 2:** Analysis of real samples and recoveries of 4-NP from different matrixes using the fluorescent HB-CNDs.

Sample	4-NP Concentration (μΜ)		Recovery (%)
	Added	Determined	
Effluent from a wastewater treatment plant	0	non-detected	-
	7.5	7.6	101.3
	15.4	16.1	104.5
	22.6	22.2	98.2
Human urine	0	non-detected	-
	7.5	7.8	104.0
	15.4	15.3	99.3
	22.6	23.2	102.7

## Data Availability

Data are available upon request.

## Supplementary Materials

The following supporting information can be downloaded at https://www.mdpi.com/article/10.3390/nano13101689/s1, Table S1: % carbon and nitrogen components and % atomic concentration of the CNDs. Figure S1: Size distribution analysis of CNDs by DLS. Figure S2: TEM images of CNDs. A. Hydrophilic, B. Hydrophobic. Figure S3: UV-Vis absorption spectra of (a) HL-CNDs and (b) HB-CNDs. Figure S4: Quenching of the emitted fluorescence of the HL-CNDs at various pH values (λ_ex_ = 330 nm). Figure S5: Percentage of extraction of 4-NP in different organic solvents. Figure S6: Percentage of extraction of 4-NP in ethyl acetate at various pH values. Figure S7: Quenching of the emitted fluorescence of HL-CNDs caused by different concentrations of 4-NP under optimum conditions. Figure S8: UV-Vis absorption spectra of HL-CNDs, 4-NP and their mixture (real and algebraic). Figure S9: Normalized excitation and emission spectra of HL-CNDs and absorption spectrum of 4-NP. Table S2: Reported fluorescent dots used as probes for the detection of 4-NP. References [41,42,43,44,45] are cited in the Supplementary Materials.
