# Supplementary material for: Two-Birds-with-One-Stone Synthesis of Hydrophilic and Hydrophobic Fluorescent Carbon Nanodots from Dunaliella salina Biomass as 4-Nitrophenol Nanoprobes Based on Inner Filter Effect and First Derivative Redshift of Emission Band
Source: Nanomaterials (Basel). 2023 May 21;13(10):1689. doi: 10.3390/nano13101689 (PMC10223520; doi:10.3390/nano13101689)
Supplement: Supplementary file 1 [file nanomaterials-13-01689-s001.zip › nanomaterials-2398390-supplementary.pdf]

Supplementary Materials

Two-Birds-with-One-Stone Synthesis of Hydrophilic and Hydrophobic Fluorescent Carbon Nanodots from *Dunaliella salina* Biomass as 4-Nitrophenol Nanoprobes Based on Inner Filter Effect and First Derivative Redshift of Emission Band

Thomais A. Skolariki [1], Theodoros G. Chatzimitakos [1], Lamprini Sygellou [2] and Constantine D. Stalikas [1,*]

[1] Laboratory of Analytical Chemistry, Department of Chemistry, University of Ioannina, 45110 Ioannina, Greece; thomiskolariki@windowslive.com (T.A.S.); chatzimitakos@outlook.com (T.G.C.)
[2] Foundation for Research and Technology Hellas/Institute of Chemical Engineering Sciences (FORTH/ICE-HT), Stadiou Str., P.O. Box 1414, 26504 Rio-Patras, Greece; sygellou@iceht.forth.gr
* Correspondence: cstalika@uoi.gr

Instrumentation and characterization

All fluorescence measurements were performed on an FS5 spectrofluorometer (Edinburgh Instruments) with an excitation and emission slit set at 5 nm bandpass in a 1 cm × 1 cm quartz cell. The UV/Vis absorption spectra were recorded on a Lambda 35 UV/VIS spectrometer (Perkin Elmer, Germany). The solvothermal synthesis of CNDs was conducted using a stainless-steel Teflon-lined autoclave and a laboratory furnace while ultrasonication and centrifugation were carried out at an Emmi-D30 ultrasonicator (160 W, Emag, Germany) and a PrO-Research centrifuge (Centurion, Sci., West Sussex, UK), respectively. Lyophilization of HL-CNDs was performed at an Alpha 1-4 LD freeze-dryer (Christ, Germany). The FTIR spectra of the synthesized CNDs were recorded on a Spectrum Two FTIR using an attenuated total reflectance accessory (PerkinElmer, MA, USA). The surface analysis measurements were performed in a UHV chamber (P~5×10^{-10} mbar) equipped with a SPECS Phoibos 100-1D-DLD hemispherical electron analyzer and a non-monochromatized dual-anode Mg/Al x-ray source for XPS. The XP Spectra were recorded with AlKa at 1486.6 eV photon energy and an analyzer pass energy of 10 eV giving a Full Width at Half Maximum (FWHM) of 0.85 eV for the Ag3d5/2 line. The analyzed area was a spot of 3mm diameter. For spectra collection and treatment, including fitting, the commercial software SpecsLab Prodigy (by Specs GmbH, Berlin) was

used. The atomic ratios were calculated from the intensity (peak area) of the XPS peaks weighted with the corresponding relative sensitivity factors (RSF). The samples were in powder form and the XPS measurements were pressed in foil. Hydrodynamic diameter and zeta potential were recorded on a Zetasizer Nano Zs (Malvern Instruments Ltd., UK).

Quantum yield (QY)

Fluorescence quantum yield (QY) of the synthesized CNDs was estimated by using a previously reported method [1]. A standard solution of quinine sulfate was prepared (0.1 M H_2SO_4) [refractive index (η_2) = 1.33] and used, whose QY is reported to be 54% at 360 nm. QY was calculated with the following equation:

$$\phi_1 = \frac{\phi_2 I_1 A_2 \eta_1^2}{I_2 A_1 \eta_2^2}$$

where, I_1 and I_2 are the fluorescence intensities of the CNDs and the standard, and A_1 and A_2 are the absorbances of the CNDs and the standard solution, respectively. Finally, η_1 and η_2 are the refractive indices of the CNDs solution and the standard, respectively. HL-CNDs dispersions were prepared in water, while HB-CNDs were dispersed in ethyl acetate [refractive index (η_1) = 1.33 and1.37, respectively]. The absorbances of the solutions were measured at the excitation wavelength, using a UV-Vis spectrophotometer. To reduce reabsorption effects, absorbance rates of the individual solutions in cuvettes were maintained below 0.1 at the excitation wavelength.

Characterization of CNDs

Table S1: % Carbon and nitrogen components and % atomic concentration of the CNDs.

sample	% carbon components				
	C-C sp²	*C-C sp³*	*C-O(H)*	*C=O*	*COOH*
HL-CNDs	18.5	49.1	13.2	7.8	11.4
HB-CNDs	29.6	42.8	22.4	2.1	3.1
	% nitrogen components				
	C–NH–C=O			$–NH_3^+$	

HL-CNDs	100		-
HB-CNDs	90.6		9.4
	% atomic concentration of C, O, N		
	% C at.	% O at.	% N at.
HL-CNDs	*67.7 ± 0.2*	*29.1 ± 0.1*	*3.50 ± 0.06*
HB-CNDs	*84.5 ± 0.1*	*13.3` ± 0.1*	2.22 ± *0.06*

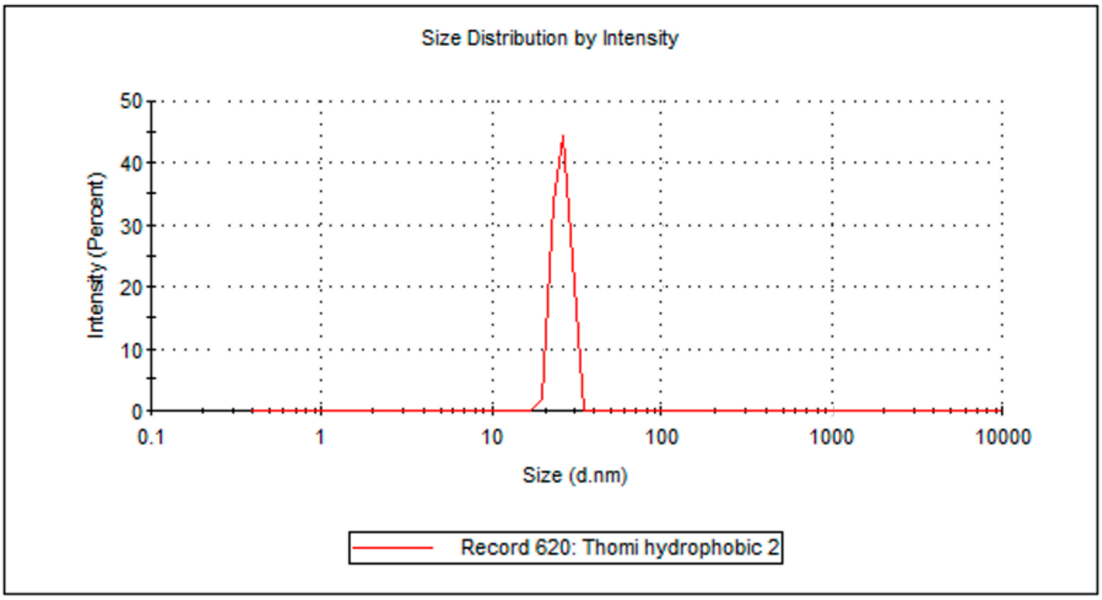

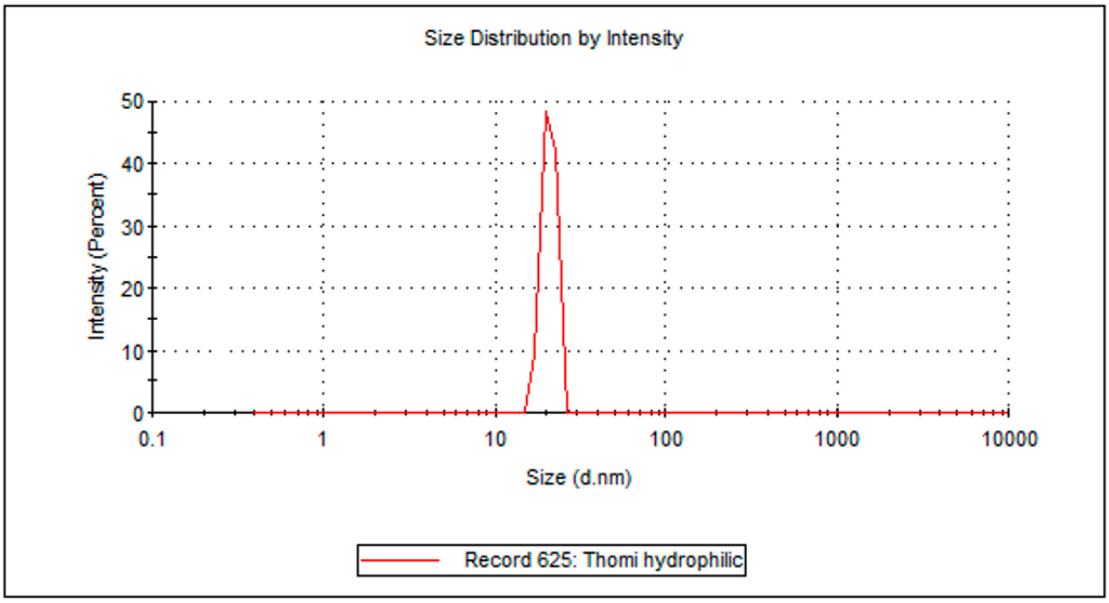

Figure S1. Size distribution analysis of CNDs by DLS.

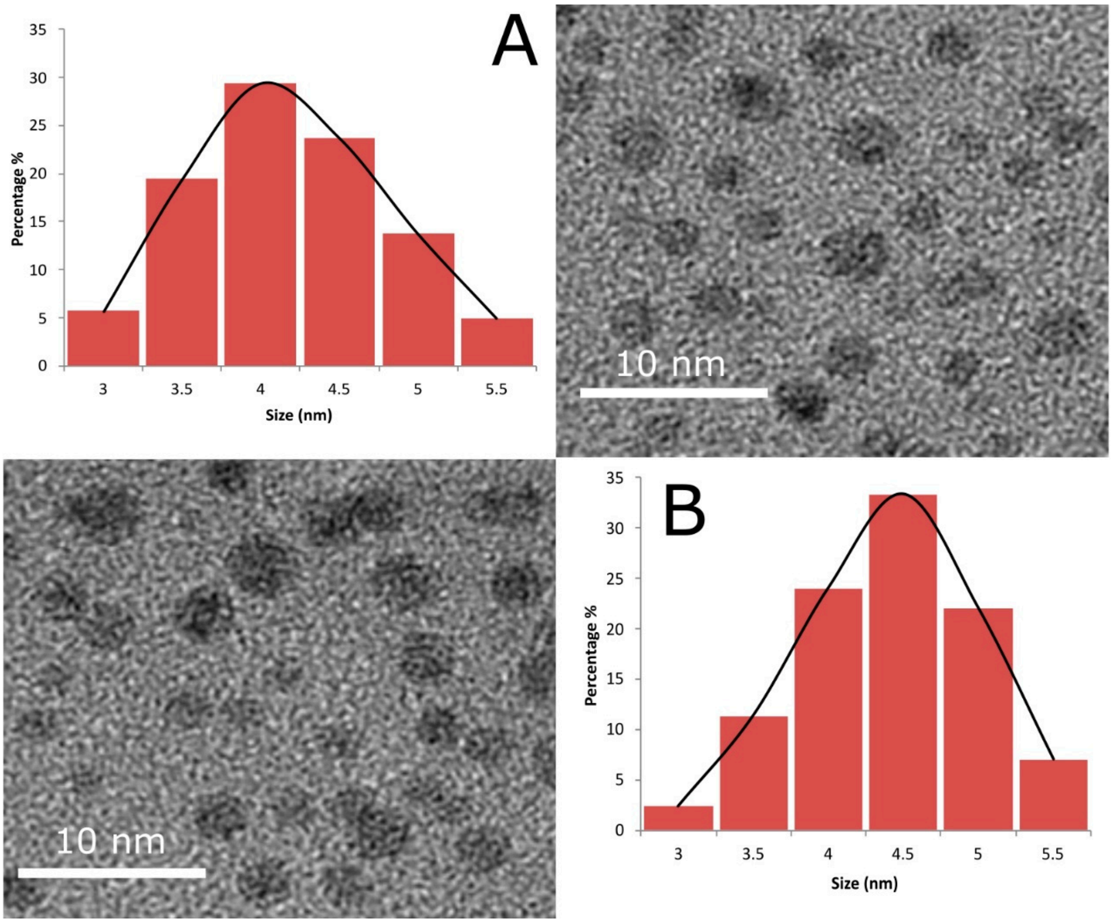

Figure S2. TEM images of CNDs. A. Hydrophilic, B. Hydrophobic.

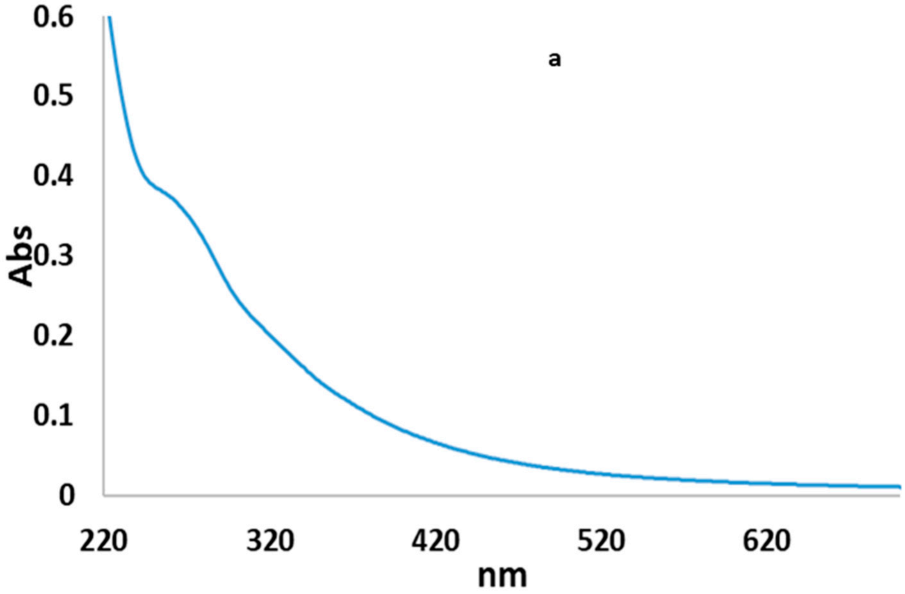

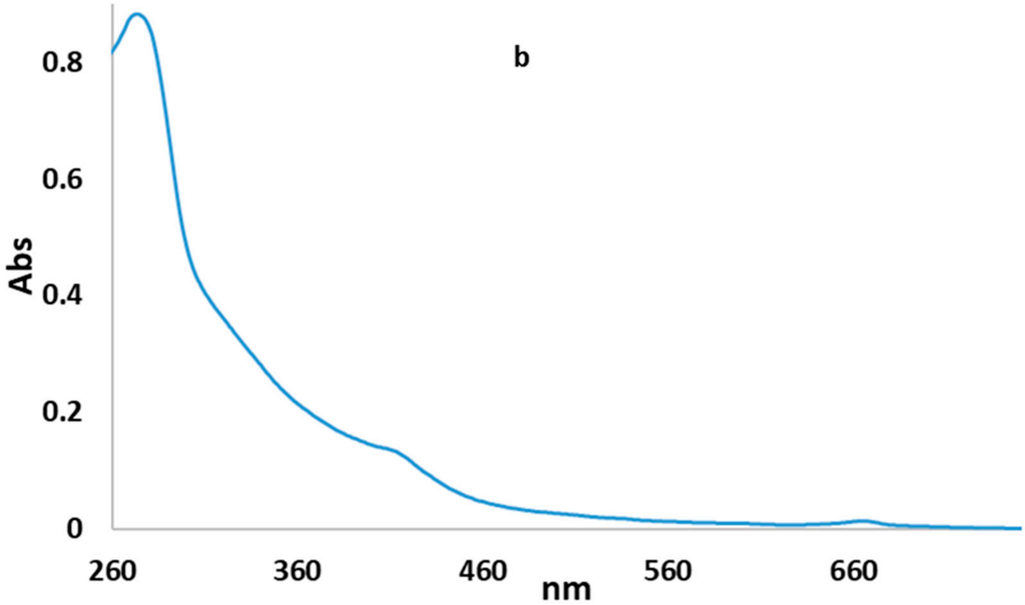

Figure S3. UV–vis absorption spectra of (a) HL-CNDs and (b) HB-CNDs

Optimization of 4-NP sensing and selectivity study

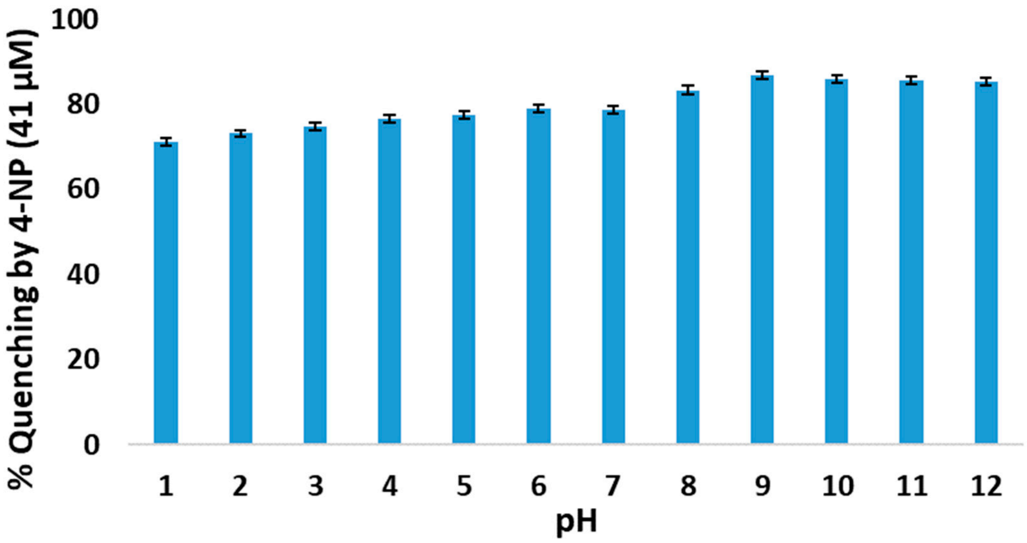

Figure S4. Quenching of the emitted fluorescence of the HL-CNDs, at various pH values (λ_{ex}=330 nm).

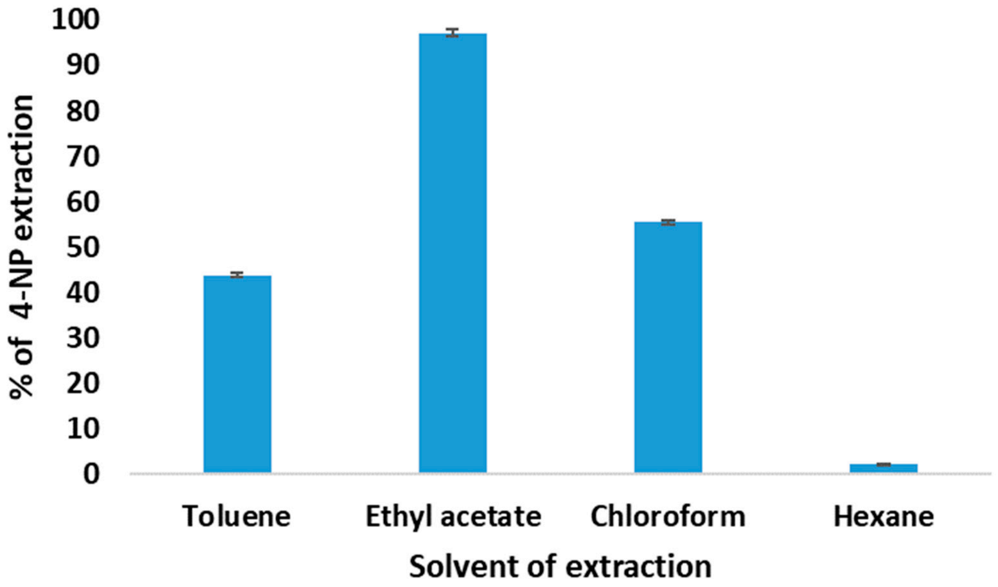

Figure S5. Percentage of extraction of 4-NP in different organic solvents.

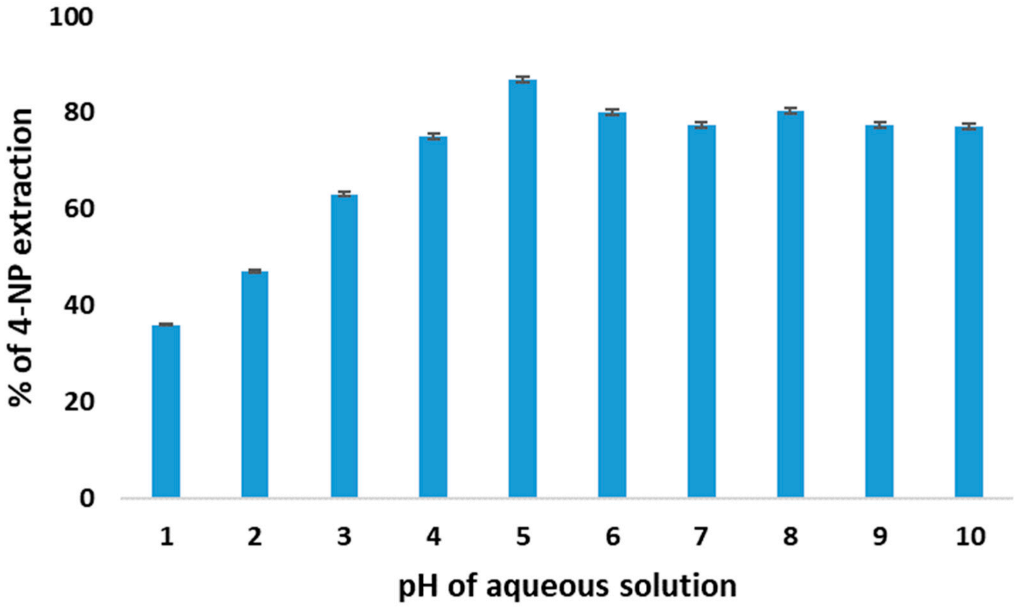

Figure S6. Percentage of extraction of 4-NP in ethyl acetate at various pH values.

Probing 4-NP with HL-CNDs

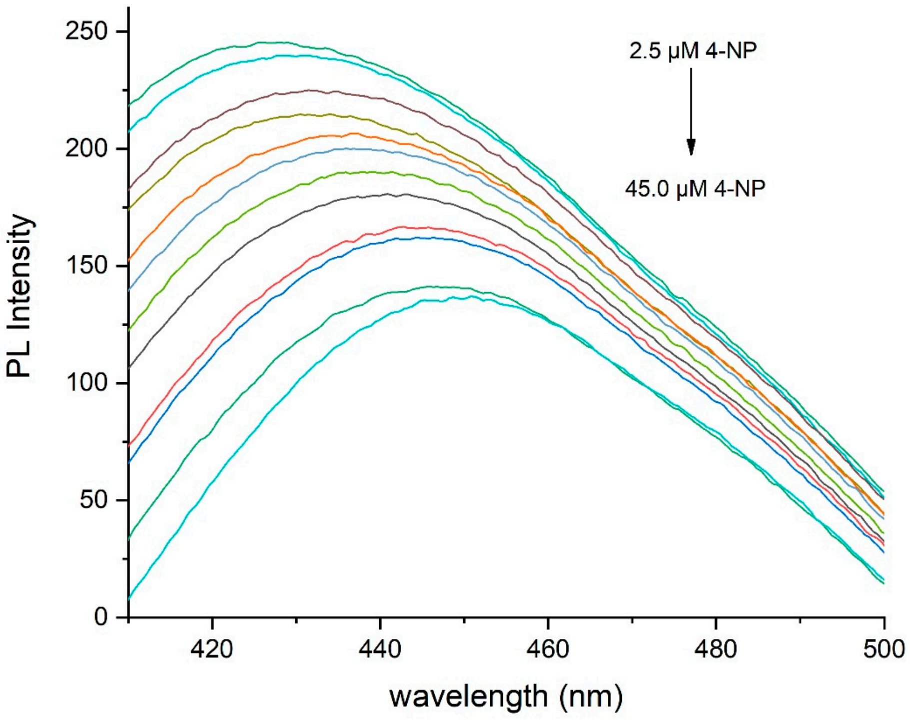

Figure S7. Quenching of the emitted fluorescence of HL-CNDs caused by different concentrations of 4-NP under optimum conditions.

Quenching mechanism of the HL-CNDs

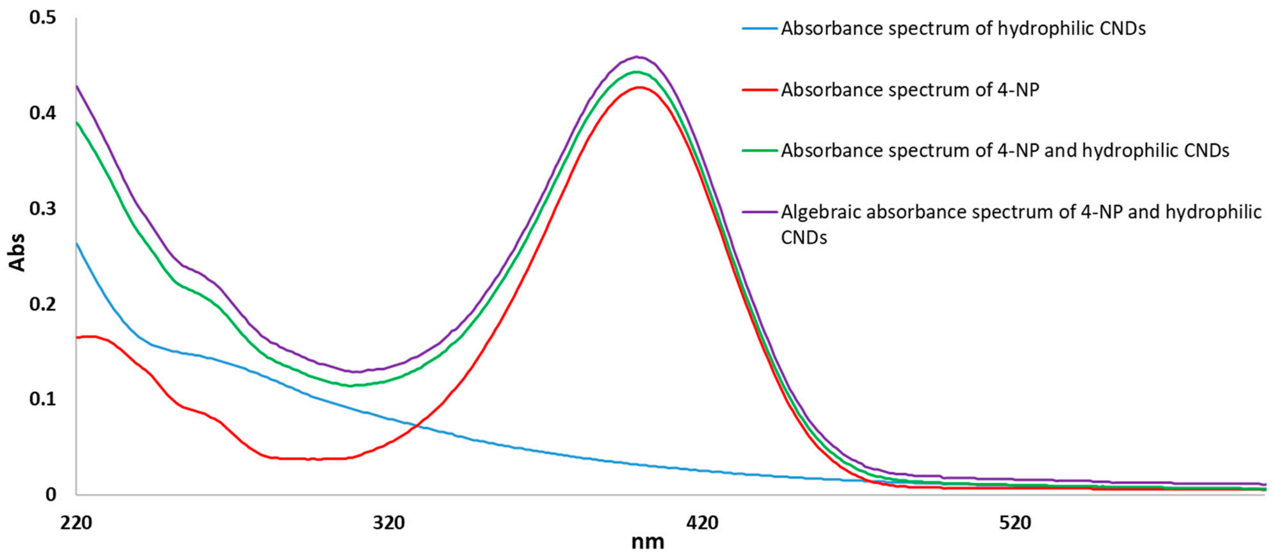

Figure S8. UV-Vis absorption spectra of HL-CNDs, 4-NP, their mixture (real and algebraic).

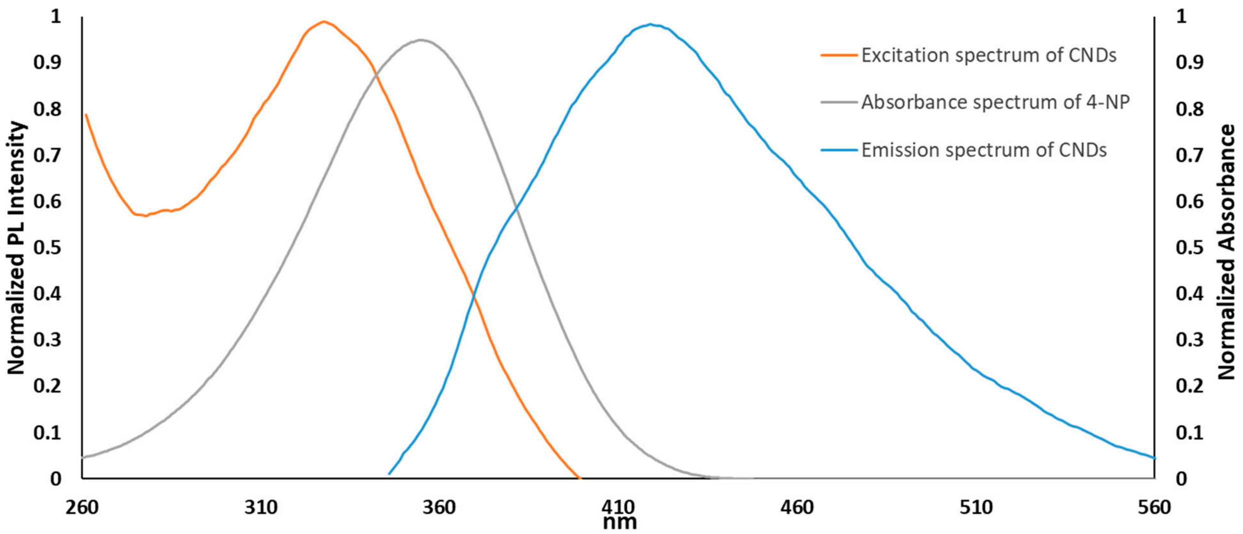

Figure S9. Normalized excitation and emission spectra of HL-CNDs and absorption spectrum of 4-NP.

Table S2: Reported fluorescent dots used as probes for the detection of 4-NP.

Materials used	Method	Matrix	Recoveries (%)	LOD (µM)	Linear range (µM)	Ref.
Citric acid and urea	Fluorescent N doped oxidized carbon dots	Tap and industrial water	data not shown	2	2-100	[2]
Graphene	Fluorescent MIP-coated GQDs	Tap water and river water	97.0-100.5	0.06	0.14-21.6	[3]
Polyethylene glycol and sulfur powder	Fluorescent sulfur quantum dots	Tap, river mineral water	90.9-102	0.07	0.2-30 and 30-90	[4]
Hexamethylenetetramine and ethanediamine	Fluorescent N-doped carbon dots	River, industrial water	98.0-106.7	0.20	0.5-70	[5]
Dunaliella salina	Fluorescent hydrophilic carbon dots	Tap water, effluent water from a wastewater treatment plant, human urine	102.2-113.7	0.3	0.8-45.0	This work
Dunaliella salina	Fluorescent hydrophobic carbon dots	Effluent water from a wastewater treatment plant, human urine	98.2-104.5	0.5	1.4-23.0	This work
Dunaliella salina	Fluorescent hydrophilic carbon dots (red-shift method)	Tap water, effluent water from a wastewater treatment plant, human urine	104.6-111.6	4.2	5.0-45.0	This work

References

[1] C. Jiang, H. Wu, X. Song, X. Ma, J. Wang, M. Tan, Presence of photoluminescent carbon dots in Nescafe® original instant coffee: Applications to bioimaging, Talanta. **2014**, *127*, 68–74. https://doi.org/10.1016/j.talanta.2014.01.046.

[2] N.K.R. Bogireddy, R.C. Silva, M.A. Valenzuela, V. Agarwal, 4-nitrophenol optical sensing with N doped oxidized carbon dots, J. Hazard. Mater. **2019**, *386*, 121643. https://doi.org/10.1016/j.jhazmat.2019.121643.

[3] Y. Zhou, Z. bei Qu, Y. Zeng, T. Zhou, G. Shi, A novel composite of graphene quantum dots and molecularly imprinted polymer for fluorescent detection of paranitrophenol, Biosens. Bioelectron. **2014**, *52*, 317–323. https://doi.org/10.1016/j.bios.2013.09.022.

[4] X. Peng, Y. Wang, Z. Luo, B. Zhang, X. Mei, X. Yang, Facile synthesis of fluorescent sulfur quantum dots for selective detection of p-nitrophenol in water samples, Microchem. J. **2021**, *170*, 106735. https://doi.org/10.1016/j.microc.2021.106735.

[5] S. Liao, Z. Ding, S. Wang, F. Tan, Y. Ge, Y. Cui, N. Tan, H. Wang, Fluorescent nitrogen-doped carbon dots for high selective detecting p-nitrophenol through FRET mechanism, Spectrochim. Acta - Part A Mol. Biomol. Spectrosc. **2021**, *259*, 119897. https://doi.org/10.1016/j.saa.2021.119897.
